# Efficacy of acupuncture for Parkinson’s disease with mild cognitive impairment: study protocol for a randomized controlled trial

**DOI:** 10.3389/fneur.2025.1674098

**Published:** 2025-10-24

**Authors:** Shunan Wu, Ian I. Leong, Jingqi Fan, Xin Liu, Elaine Liu, Suying Lei, Xiaoyan Xu, Kaihao Liao, Li-Xing Zhuang

**Affiliations:** ^1^First Clinical Medical College of Guangzhou University of Chinese Medicine, Guangzhou, China; ^2^The First Affiliated Hospital of Guangzhou University of Chinese Medicine, Guangzhou, China; ^3^University of Pennsylvania, Systems Pharmacology and Translational Therapeutics Department, Philadelphia, PA, United States

**Keywords:** Parkinson’s disease, mild cognitive impairment, acupuncture, protocols, randomized controlled trial

## Abstract

**Background:**

Parkinson’s disease with mild cognitive impairment (PD-MCI) is one of the primary non-motor symptoms of Parkinson’s disease (PD). PD-MCI represents an early stage of cognitive impairment in PD and serves as a potential precursor to PDD. To date, research on the treatment of mild cognitive impairment in PD remains limited. Acupuncture, a classical therapeutic modality in Traditional Chinese Medicine, exhibits superior therapeutic outcomes to pharmacotherapy for mild cognitive impairment while avoiding drug-associated adverse effects.

**Objective:**

This randomized, single-blind clinical trial aims to evaluate the efficacy and safety of acupuncture for PD-MCI. To improve the credibility of acupuncture research evidence through employing a sham acupuncture device as a control.

**Methods and analysis:**

This is a prospective, sham-controlled, subject-blinded and assessor-blinded trial, which conducted at a single center in China. A total of 72 eligible PD-MCI volunteers will be randomized using a simple randomization method in a 1:1 ratio into the acupuncture group and the placebo acupuncture group to receive either acupuncture or placebo acupuncture for 20 sessions over a succession of 5 weeks. The primary outcome measure will be the Montreal Cognitive Assessment Scale (MoCA) score. The secondary outcome measures will be the scores of Mini-Mental State Examination (MMSE), Unified Parkinson’s Disease Rating Scale Motor Examination (UPDRSIII) and the level of neurofilament light polypeptide (NfL) and glial cell-derived neurotrophic factor (GDNF). The evaluation will be assessed before and after treatment.

**Discussion:**

This study represents the first randomized, single-blind clinical trial investigating acupuncture treatment for cognitive dysfunction in Parkinson’s disease. An auxiliary device designed by our team, featuring a flat-head needle and adjustable sleeve, will be used for placebo acupuncture procedure to achieve a single-blind effect. Serum NfL and GDNF levels will be incorporated to elucidate the mechanisms underlying acupuncture’s effects and explore specific biomarkers of PD. The aim of this study is to provide reliable clinical evidence for the treatment of PD-MCI and improve patients’ survival and quality of life.

**Clinical trial registration:**

https://www.chictr.org.cn/, identifier ChiCTR2400082082.

## 1 Introduction

Parkinson’s disease (PD) represents a progressive neurodegenerative disorder primarily affecting individuals aged 50 years and older. While its clinical manifestations are mainly characterized by bradykinesia, resting tremor, and rigidity, the disease’s substantial burden also often stems from its non-motor symptoms such as mental disorders, cognitive impairment, sleep disorders, and sensory disorders. Among these, Parkinson’s disease with mild cognitive impairment (PD-MCI) has emerged as a particularly significant concern, affecting 30–40% of PD patients and progressing to Parkinson’s disease dementia (PDD) at an annual rate of 6–15% (1). PD-MCI as both a clinical precursor and modifiable risk factor for PDD (2), highlighting the critical importance of early detection and intervention.

At present, the pathogenesis of PD-MCI remains incompletely understood. Relevant studies suggest the involvement of neurotransmitter system impairments, such as dopaminergic and cholinergic systems (3, 4). In clinical practice, while cholinesterase inhibitors and dopamine receptor agonists constitute the mainstay of PD-MCI pharmacotherapy, their clinical benefits remain suboptimal and are frequently accompanied by adverse effects. Therefore, more treatment options for PD-MCI need to be explored.

Acupuncture, as a well-established therapeutic approach in Traditional Chinese Medicine (TCM), demonstrates clinical advantages including procedural simplicity and favorable safety characteristics. It has gained widespread clinical adoption as a non-pharmacological therapy. In recent years, many researches show that acupuncture can modulate the brain activities through Functional magnetic resonance imaging (fMRI) and EEG (5–7). It was proved that acupuncture promotes cognitive function and affords neuroprotective effects against inflammation in animal experiment (8). Accumulated clinical evidence indicates that acupuncture can improve cognitive function by regulating neurotransmitters, enhancing neurotrophic factor signaling, and reducing cell apoptosis (9). More importantly, it was demonstrated that acupuncture treatment could ameliorate the non-motor symptoms, quality of life and activities of daily living in PD patients (10).

This randomized controlled trial is designed based on previous acupuncture research. It aims to evaluate the cognitive function improvement in PD-MCI patients receiving either verum or placebo acupuncture. In this study, we focus on the clinical efficacy of acupuncture for PD-MCI in order to provide an evidence-based assessment of acupuncture’s therapeutic value. We hope that we can expand the clinical treatment option for PD-MCI through the study, help to delay the disease progression, and enhance the quality of life of PD patients.

## 2 Materials and methods

This study will be a prospective, single-blind, randomized controlled clinical trial targeting Chinese PD-MCI patients. The trial will be conducted at the First Affiliated Hospital of Guangzhou University of Chinese Medicine.

The ethics committee of the First Affiliated Hospital of Guangzhou University of Chinese Medicine has approved the research protocol, which will follow the Declaration of Helsinki. The protocol has also been registered in the Chinese Clinical Trial Registry (ChiCTR2300074675).

**Figure 1:**
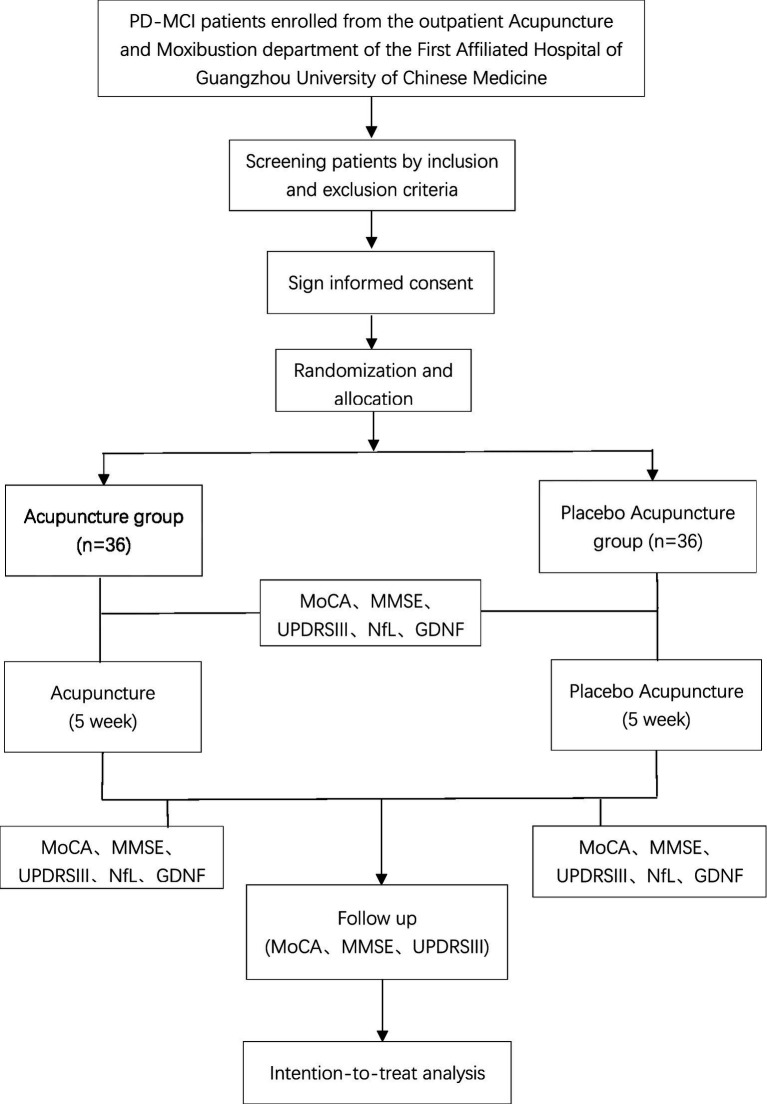
Flowchart of the trial.

A total of 72 eligible PD-MCI volunteers will be included in this study, and the flowchart is shown in Figure 1.

### 2.1 Diagnostic criteria

Diagnostic criteria for PD refer to the Movement Disorder Society Clinical Diagnostic Criteria for PD (2015) (11). The diagnostic criteria for mild cognitive impairment in PD refer to the Diagnostic Criteria for Mild Cognitive Impairment in Parkinson’s Disease formulated by the International Association of Dyskinesia in 2012 (12).

#### 2.1.1 Inclusion criteria

1. Meet the diagnostic criteria for idiopathic PD and PD-MCI.
2. Meet the diagnostic criteria of TCM tremor syndrome and stupidness.
3. Patients aged 50–70 years.
4. MoCA scale score range of 18 to 25.
5. MMSE scale score range of 21 to 26.
6. Hoehn-Yahr staging scale staged 1–3.
7. Do not participate in other drugs, acupuncture and other treatments or clinical trials within 1 month.
8. Voluntarily join the study and sign the informed consent.

#### 2.1.2 Exclusion criteria

The exclusion criteria are as follows:

1. Do not meet the diagnostic criteria and inclusion criteria.
2. Users of pharmaceutical products improving cognitive function, such as Donepezil, galantamine, memantine hydrochloride, etc.
3. Diagnosed with severe mental complications such as major depression and schizophrenia.
4. Cognitive dysfunction caused by other diseases.
5. Had participated in other drug or acupuncture studies within 30 days of treatment initiation.
6. Diagnosed with other severe illnesses such as stroke, hematopoietic system diseases, malignant tumors, etc.
7. Allergic or intolerant to acupuncture.

### 2.2 Sample size

Aligned with the study’s objective, Montreal Cognitive Assessment Scale (MoCA) was chosen as the primary outcome. According to the results of previous experiments and literature review (13, 14), the mean and standard deviation of MoCA scale in PD-MCI patients received acupuncture combined with anti-PD drug treatment were 25.32 and 4.34, while there were 21.48 and 4.44, respectively, in PD-MCI patients who received sham acupuncture combined with anti-PD drug treatment. PASS V.15.0 (NCSS, Kaysville, UT, United States) was used to calculate the sample size. We set 90% for power level and 5% for two-sided significance level. It was estimated that there were about 29 patients in each group, and considering the dropout rate of 20%, the total sample size needed for study is 72, with 36 patients in each group.

### 2.3 Randomization

Subjects with PD-MCI who met the inclusion and exclusion criteria will be randomized using a simple randomization method in a 1:1 ratio into the acupuncture group and the placebo acupuncture group. Random sequence will be generated by a third party who do not participate in the study using SPSS Statistics 26.0 (IBM SPSS Statistics Inc., Chicago, United States).

### 2.4 Blinding

To ensure the objectivity and accuracy of the results, this study adopts the single blind method to blind the subjects and the efficacy evaluator. Due to the limitations of needle operation, it is impossible to blind the operating physician, so the efficacy evaluator is a third party independent of the treatment operation and grouping to avoid subjective bias of the researcher. During the treatment, both groups of participants will be required to wear an eye mask to ensure the implementation of the blind method. At the end of the study, the blind will be uncovered and the participants will be informed of their group and acupuncture method.

### 2.5 Interventions

All the acupuncturists have obtained the doctor qualification certificate and have certain clinical experience. At the same time, before the start of the study, all treating physicians should undergo systematic training to ensure the consistency of operation. During the treatment process, each patient will be treated by the same acupuncturist throughout the entire procedure.

**Figure 2:**
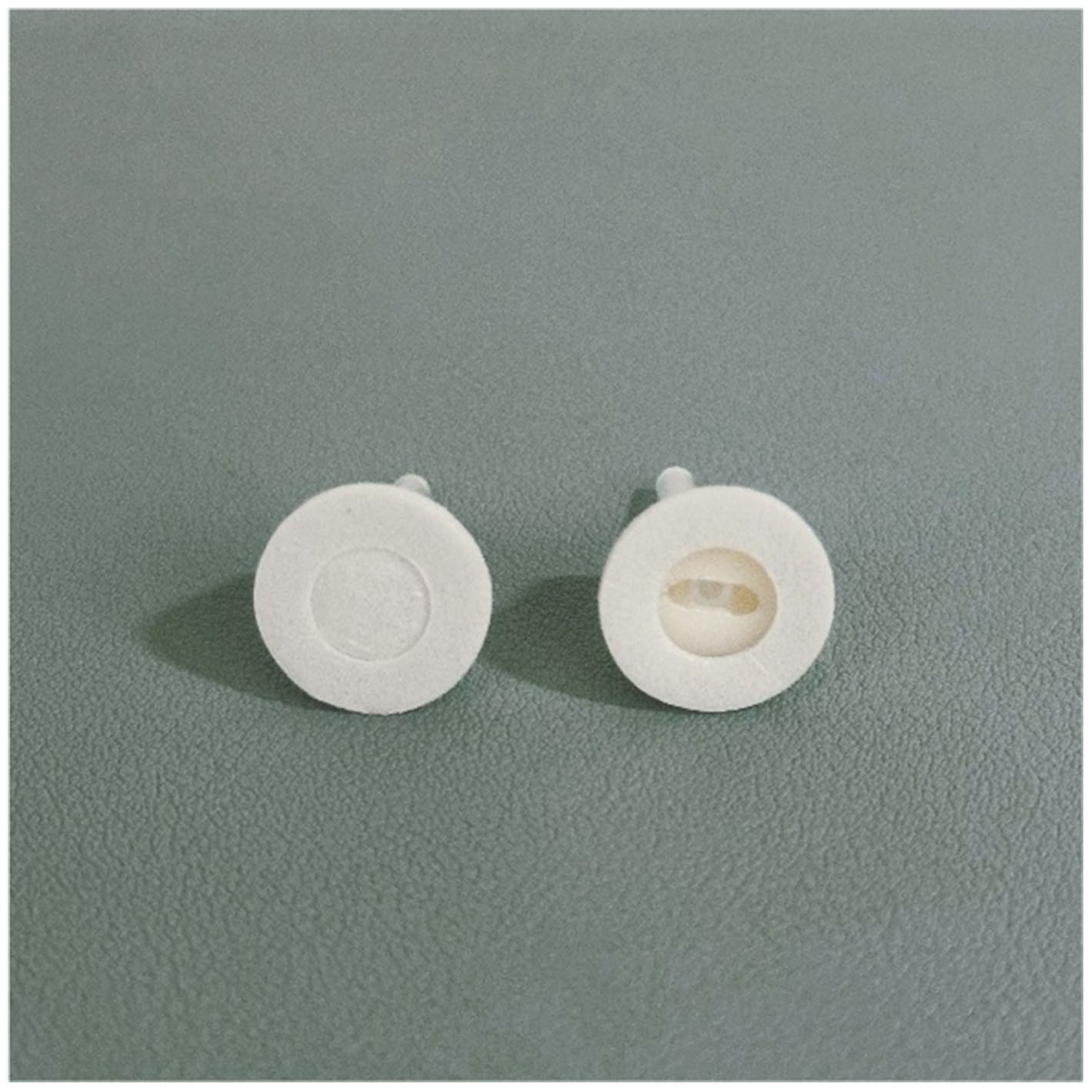
The placebo acupuncture device and the acupuncture device.

**Figure 3:**
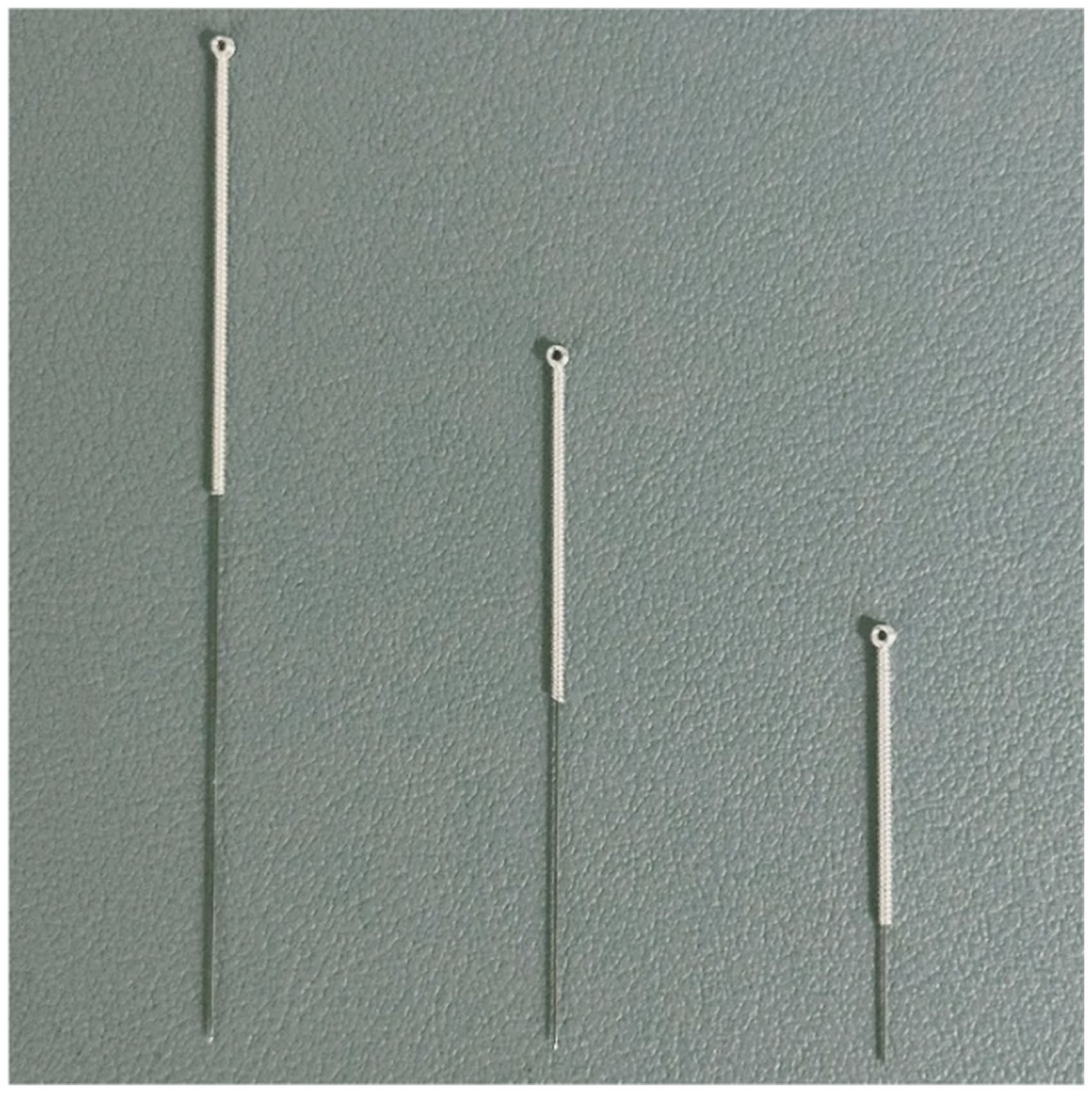
The flat head needle (40 mm, 25 mm real needle and 10 mm sham needle).

**Figure 4:**
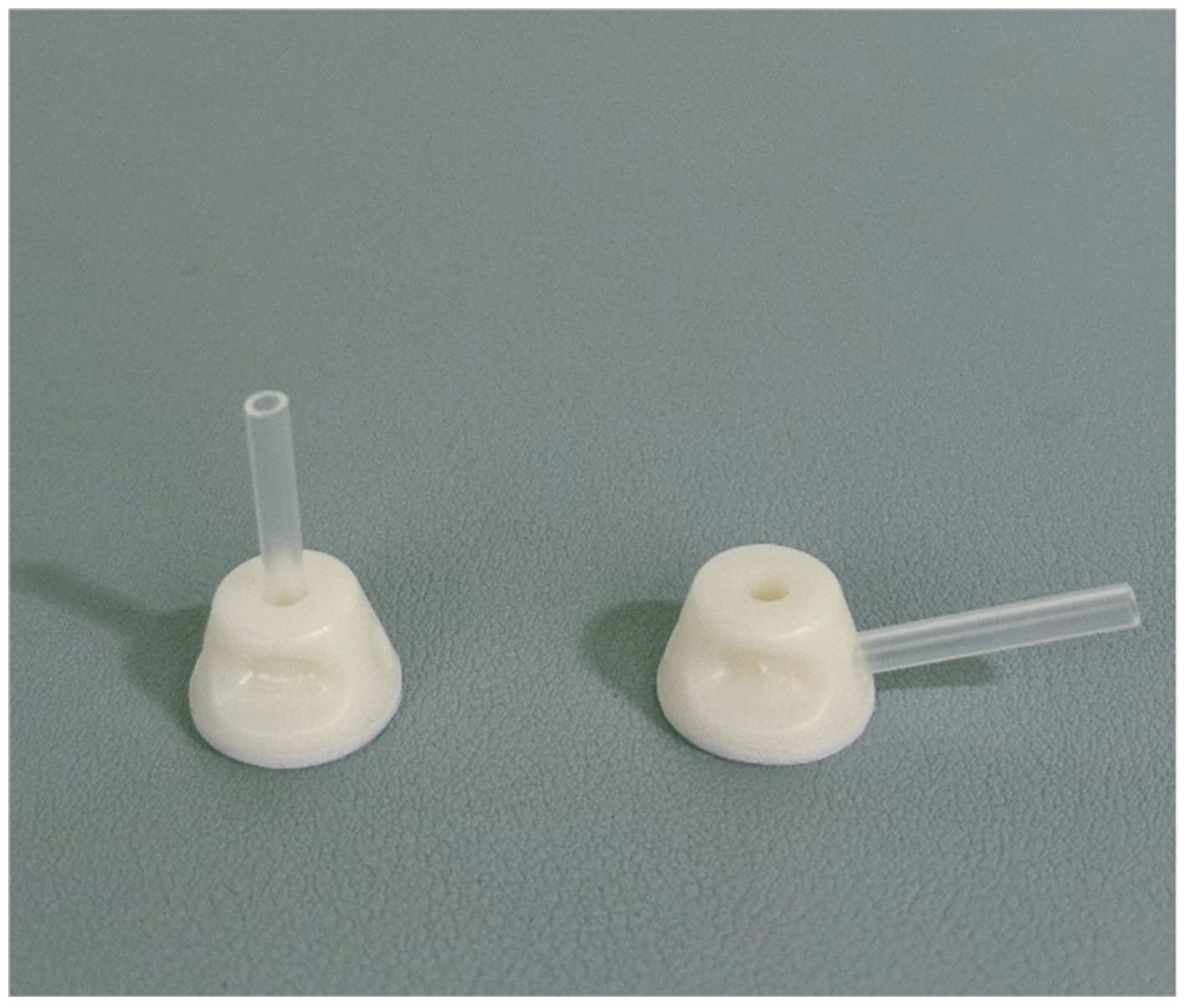
The angle of the sleeve (needle at 90-degree and 15-degree).

An auxiliary device designed by our team will be used during the acupuncture procedure in order to achieve a single-blind effect, which has passed the national utility model patent (patent number: ZL202121352221.7), and the article introducing the device has been published in the journal of Chinese Acupuncture & Moxibustion (15). This new auxiliary acupuncture device includes an acupuncture device and a placebo acupuncture device with exactly the same appearance, which consists of a base sticking to the skin and a sleeve. The base of the acupuncture device is hollow, while the placebo acupuncture device is sealed by a bonding layer (Figure 2). A flat head needle (Tianxie, Suzhou Medical Appliance Factory, Suzhou, China; 0.30 mm × 10 mm) (Figure 3) will be used for the procedure. The acupuncturist can adjust the sleeve according to the insertion angle of needles (Figure 4).

#### 2.5.1 Acupuncture group

**Figure 5:**
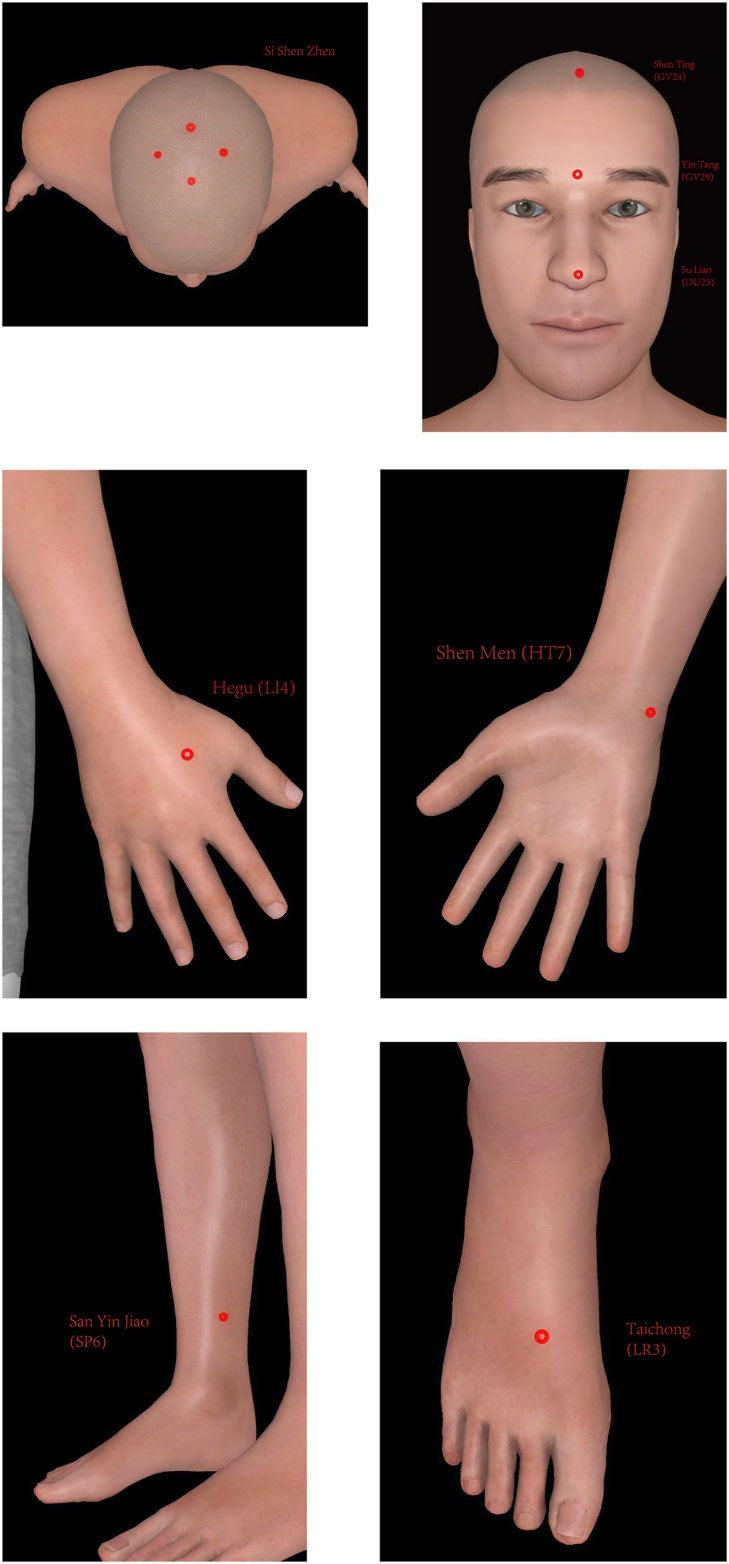
The location of each acupoint.

Patients in the acupuncture group will receive 20 sessions of acupuncture (four times a week for 5 weeks), during which the original effective dose of anti-Parkinson’s disease drugs will be maintained. If there is a need to increase or decrease the dose under special circumstances, the change will be recorded in the case report form. Acupuncture points of the acupuncture group include Si Shen Zhen (Four acupoints, consisting of GV21, GV19, and next to GV20 1.5 cun bilateral), Shen Ting (GV24), Yin Tang (GV29), Su Liao (DU25), Shen Men (HT7) (bilateral), San Yin Jiao (SP6) (bilateral), Hegu (LI4) (bilateral), and Taichong (LR3) (bilateral). The positioning of Si Shen Zhen is in accordance with the “Application of Jin San Zhen acupoint Group” (16), and other acupoints are in accordance with the National Standard of the People’s Republic of China’s (GB/T 12346–2006) standard (17). Acupuncture will be processed with disposable, sterile steel needles (Tianxie, Suzhou Medical Appliance Factory, Suzhou, China; 0.25 × 25 mm, 0.25 × 40 mm). Patients will wear the blindfold throughout the treatment. After routine skin disinfection, the acupuncturist will affix the acupuncture device (Guangzhou Suixin Medical Equipment Co., LTD.) to the acupoint skin. 0.25 × 25 mm acupuncture needles will be used for Si Shen Zhen, Shen Ting (GV24) and Yin Tang (GV29) with a 15-degree angle. 0.25 × 40 mm acupuncture needles will be used for Su Liao (DU25), Shen Men (HT7) (bilateral), San Yin Jiao (SP6) (bilateral), Hegu (LI4) (bilateral), and Taichong (LR3) (bilateral) with a 90-degree angle. The acupuncture needle is inserted through the sleeve and the perforated base into the skin. The location of each acupoint is shown in Table 1 and Figure 5. The needle will be retained for 30 min.

**Table 1:** The location of each acupoint.

Acupoint	Location
Si Shen Zhen	Four acupoints, consisting of GV21, GV19, and next to GV20 1.5 cun bilateral
Shen Ting	On the head, 0.5 cun directly above the midpoint of the anterior hairline.
Yin Tang	On the head, in the depression between the medial ends of the two eyebrows.
Su Liao	On the face, at the tip of the nose, in the exact center.
Shen Men	In the anterior wrist region, at the ulnar end of the distal wrist crease, on the radial side of the tendon of the ulnar flexor muscle of the wrist.
San Yin Jiao	On the medial side of the leg, 3 cun above the tip of the medial malleolus, posterior to the medial border of the tibia.
Hegu	On the dorsum of the hand, between the 1st and 2nd metacarpal bones, at the midpoint of the radial side of the 2nd metacarpal bone.
Taichong	On the dorsum of the foot, in the depression between the 1st and 2nd metatarsal bones, at the junction of the two bones.

#### 2.5.2 The placebo acupuncture group

Patients in the acupuncture group will receive 20 sessions of placebo acupuncture using sham acupuncture needles (four times a week for 5 weeks), during which the original effective dose of anti-Parkinson’s disease drugs will be maintained. If there is a need to increase or decrease the dose under special circumstances, the change will be recorded in the case report form. The acupuncture points of the placebo acupuncture group will be the same as those of the acupuncture group. Patients will wear the blindfold throughout the treatment. After routine skin disinfection, the acupuncturist will affix the placebo acupuncture device (Guangzhou Suixin Medical Equipment Co., LTD.) to the acupoint skin. The acupuncturist adjusts the sleeve according to the insertion angle of needles. The flat head needle (Tianxie, Suzhou Medical Appliance Factory, Suzhou, China; 0.30 mm × 10 mm) will be used for the procedure, and will be inserted through the cannulas to make patients feel slightly needled. The needle will be maintained for 30 min.

### 2.6 Outcome measure

General information of the participants, including name, age, sex, disease duration, education, Levodopa-equivalent dose, will be collected before the study begin. Primary outcome measure will be the Montreal Cognitive Assessment Scale (MoCA). The secondary outcome will be the Mini-Mental State Examination (MMSE), Unified Parkinson’s Disease Rating Scale Motor Examination (UPDRSIII), serum neurofilament light polypeptide (NfL) and serum glial cell-derived neurotrophic factor (GDNF). Serological indicators will be measured by enzyme-linked immunosorbent assay (ELISA). The schedule of the outcome measurements is shown in Table 2.

**Table 2:** Schedule of enrollment, intervention, assessment and safety.

Study period								
Time point (Week)	−1	0	1	2	3	4	5	25
Enrollment								
Eligibility screen	×							
Informed consent	×							
Randomization		×						
Allocation		×						
Intervention								
Acupuncture			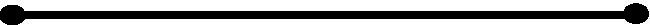					
Placebo Acupuncture			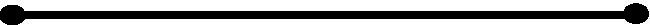					
Assessments								
MoCA		×					×	×
MMSE		×					×	×
UPDRSIII		×					×	×
NfL		×					×	
GDNF		×					×	
Safety								
Vital signs		×		×			×	×
Adverse events			×	×	×	×	×	×

#### 2.6.1 Primary outcome

##### 2.6.1.1 The Montreal cognitive assessment scale (MoCA)

The MoCA has a certain sensitivity to screening PD-MCI, and the content of the scale is divided into 8 cognitive domains, including visuospatial and executive, naming, memory, attention, language, abstract ability, delayed recall, and orientation. The total score is 30 points. A score above 26 is normal, and a score between 18 and 25 refers to mild cognitive impairment. If when using this table, Add 1 point as correction when patients have less than 12 years of education.

#### 2.6.2 Secondary outcomes

##### 2.6.2.1 Mini-mental state examination (MMSE)

MMSE includes five cognitive areas of orientation, memory, attention and calculation, recall ability, and language ability. The total score is 30, and the score between 21 and 26 is considered mild cognitive impairment.

##### 2.6.2.2 Unified Parkinson’s disease rating scale motor examination (UPDRSIII)

The UPDRS is a commonly used scale for the overall evaluation of PD patients and consists of four parts. UPDRSIII mainly evaluates the motor function of the patients. There are 14 items in total, the highest score of each item is 4 points, and the total score is 56 points. The higher the score, the more serious the motor symptoms of PD patients.

The above scales will be assessed before and after treatment.

##### 2.6.2.3 Blood serum index

Cognitive impairment in PD is associated with dysfunction of a variety of neurotransmitter systems, including acetylcholinergic, dopaminergic, norepinephrine and 5-hydroxytryptaminergic. When the axons of nerve cells are damaged, they release serum neurofilament light polypeptide (NfL) into the cerebrospinal fluid and eventually into the blood (18). Serum glial cell-derived neurotrophic factor (GDNF) promotes the survival of dopaminergic (DA) neurons in the substantia nigra. GDNF may act in combination with neurotransmitters (HVA, 5-HT, GABA and Ach) to affect attention, memory and executive function in PD-MCI patients (19). Therefore, NfL and GDNF levels can reflect the activity of brain neurons, and NfL and GDNF can be used as biomarkers related to cognitive function.

Serum concentrations of NfL and GDNF will be measured before and at the end of treatment using enzyme-linked immunosorbent assay (ELISA), which follows the kit instructions strictly. Both ELISA kits will be provided by Jiangsu Enzyme Free Industrial Co., LTD.

### 2.7 Safety evaluation

During the implementation of the study, adverse events of acupuncture, such as infection, bleeding, fainting, broken needles and subcutaneous hematoma will be handled promptly and recorded in the Adverse Event record Form. In case of serious adverse events, acupuncture treatment will be stopped and timely treatment measures will be carried out immediately. We will report to the Ethics Committee of the First Affiliated Hospital of Guangzhou University of Chinese Medicine at the same time. All adverse events will be classified and reported following CONSORT harm guideline (20).

### 2.8 Follow-up

Patients will be followed up 6 months after the end of treatment. The outcomes scales will be assessed to evaluate the long-term efficacy of acupuncture.

### 2.9 Data management and quality control

To ensure trial consistency and accuracy, all team members will receive standardized training prior to study initiation. All members will receive training on the localization of acupoints, the application of sham needles, the evaluation of assessment scales, patient communication, as well as the methods and time points for follow-up. The researcher responsible for the assessment needs to be proficient in the questions and operations in the case report form (CRF), conduct an objective assessment of the subjects, and both the assessor and the subjects need to sign the CRF to ensure the reliability and integrity of the data. Researchers will strictly keep the case observation forms and set access permissions for electronic data, allowing only authorized personnel to enter, modify and view the data to ensure data security and protect the privacy of the subjects. Data quality will be monitored regularly to check whether the data are missing, wrong or invalid, and to ensure the completeness and rationality of the data.

### 2.10 Statistical analysis

In this study, IBM SPSS Statistics 26.0 statistical software will be used to analyze the collected clinical data. Shapiro–Wilk will be used to test the normality before statistical analysis. Counting data is expressed as a percentage using a chi-square test. The measurement data meeting the normal distribution will be represented by mean ± standard deviation, while those not meeting the normal distribution will be represented by quartile. The independent sample t test will be used to compare the two groups with normal distribution and homogeneous variance, and the Mann–Whitney U test will be used to compare the two groups with non-normal distribution. The comparison difference before and after treatment will be treated by paired sample t test with normal distribution, and Wilcoxon rank sum test with abnormal distribution. The two groups of repeated measurement data do not meet the normal distribution of the generalized estimation equation, the simple effect in the group will be analyzed by Friedman test. Two-sided test will be applied uniformly in this study, and *p* ≤ 0.05 will be the standard of statistical significance.

To evaluate the clinical relevance of the findings, we will perform a responder analysis based on the minimal clinically important difference (MCID) for the MoCA. Based on previous literature in cognitive impairment (21), a change of ≥1.22 points on the MoCA scale will be considered clinically meaningful. Participants who show an improvement of ≥1.22 points from baseline to post-treatment will be classified as ‘responders’. The difference in the proportion of responders between the study groups will be compared using a Chi-square test, with a *p*-value < 0.05 considered statistically significant.

## 3 Discussion

Parkinson’s disease with mild cognitive impairment (PD-MCI) is a primary non-motor symptom of PD. Recent studies have demonstrated widespread cognitive decline among PD patients, often accompanied by more severe depression compared to those without cognitive impairment (22). Additional research indicates that PD-MCI patients have a 39–59% probability of progressing to Parkinson’s disease dementia (PDD) within 5 years (23). This suggests that PD-MCI represents an early stage of cognitive impairment in PD and a probable transitional stage preceding PDD. Therefore, preventing factors that contribute to the conversion from PD-MCI to PDD is of significant importance in delaying the onset of PDD. Additionally, cognitive dysfunction in Parkinson’s disease is associated with the progression of motor dysfunction (24). Improving cognitive function can contribute to the amelioration of motor symptoms in patients (25), highlighting the importance of early intervention for cognitive impairment.

This study represents the first clinical trial investigating the efficacy of acupuncture therapy for cognitive dysfunction in PD. Currently, research on the treatment of mild cognitive impairment in PD remains limited. The pathogenesis of PD-MCI has not yet been fully elucidated, and western medicine lacks targeted therapeutic approaches. Clinical trials of pharmacological interventions have failed to demonstrate consistent or sustained efficacy. The drugs treating cognitive dysfunction mostly accompanied by adverse effects such as nausea, vomiting, decreased appetite, and other gastrointestinal disturbances (26, 27). Given these limitations, there is an urgent need to explore additional safe and effective therapeutic strategies for PD-MCI.

As a classical therapeutic modality in Traditional Chinese Medicine (TCM), acupuncture has demonstrated clinically meaningful efficacy in the management of post-stroke cognitive impairment (28). Notably, acupuncture exhibits superior therapeutic outcomes to pharmacotherapy for certain psychiatric disorders while avoiding drug-associated adverse effects (29, 30). Acupuncture is a well-established clinical intervention in China. Acupuncture has regionally specific, quantifiable effects on relevant brain structures (31). Acupuncture was found to activate a wide range of brain function areas and promote the neural plasticity of motor and cognitive brain regions neural plasticity (32). Studies has proved that acupuncture can improve cognitive function, restore impaired neurological function, and lower related serum factor levels (8, 33). Recent advanced works evaluate therapeutic effect of acupuncture on human brain through different brain imaging methods, including MRI, fMRI and EEG. Acupuncture may affect brain function by enhancing or inhibiting the connectivity between brain regions, leading to indirect effects on the limb system (5). Scalp EEG and Transformer elucidated the dynamic regulatory effects of acupuncture throughout the treatment process (34). Yu et al. (35) used the EEG decoder to demonstrate that needle insertion can evoke immediate tactile sensations, stimulate peripheral nerves directly and ultimately affect brain regions via central nervous system pathways. Acupuncture increases long-range connections between left and right hemispheres (36). Nierhaus et al. (37) proved that stimulation at acupuncture points is more effective than stimulation at non-acupuncture point locations measured with EEG and fMRI.

In the previous clinical research on acupuncture treatment for PD, we found that acupuncture not only improved Parkinson’s anxiety, but also enhanced the Unified Parkinson’s Disease Rating Scale (UPDRS) scores of patients (38). UPDRS is a commonly used scale for evaluating PD patients’ quality of life, which can objectively evaluate the mental, emotional, daily living ability, motor symptoms and other aspects of PD patients. These findings suggest acupuncture has an overall improvement on the emotional and cognitive functions of Parkinson’s patients. Yan’s study (39) proved that acupuncture can significantly improve the sleep quality of PD patients. Li′s research (40) found that acupuncture treatment for constipation in PD is effective and safe. Xia et al. (41) using electroacupuncture to treat PD combined with depression can improve depressive symptoms, increase the level of BDNF in serum, and the therapeutic effect is superior to that of drug treatment alone. All research above further supports acupuncture’s multimodal benefits for PD non-motor symptoms. Regarding cognitive dysfunction specifically, both clinical studies (42, 43) and preclinical models (44, 45) across various etiologies have consistently shown acupuncture’s therapeutic potential. Therefore, based on the clinical efficacy of acupuncture in treating non-motor symptoms of PD and cognitive dysfunction in previous studies, this study will adopt acupuncture to investigate cognitive dysfunction in PD.

When it comes to acupoint selection, the acupoints located on the head like “Baihui” acupoint and “Shen Ting” acupoint are the most frequently used acupoints for enhancing cognitive performance (46, 47). We use “Si Shen Zhen,” which are next to “Baihui” 1.5 cun bilateral, and add the acupuncture point “Yin Tang” and “Su Liao” to expand the brain stimulus area. The researches showed that acupoints located at the extremities (“Shen Men,” “Hegu,” “San Yin Jiao,” “Taichong”) can stimulate cognitive, emotional brain regions, central nervous system and enhance cerebral blood circulation (48), effectively manage various health conditions (49). Therefore, this study incorporates acupoints from both the head and limbs to achieve holistic improvement.

Recent years have witnessed a growing number of clinical studies investigating acupuncture therapy. However, high-quality clinical research demands more rigorous control designs to examine potential placebo effects in acupuncture interventions. To enhance blinding effectiveness, this study employed a novel sham acupuncture device specifically designed and developed by our research team (15). The device has the advantages of adjustable angle and high stability, which can be applied to both real and sham acupuncture procedures. The special flat-head needle is used on the needle tool, and the appearance of the needle handle is consistent with that of the ordinary acupuncture needle. The device has been extensively utilized in our team’s clinical trials (50–57), with validation studies confirming its excellent blinding efficacy and yielding significant research outcomes.

To comprehensively evaluate acupuncture’s impact on the nervous system, we incorporated serum measurements of NfL and GDNF levels as secondary efficacy indicators. Both NfL and GDNF are closely related to neural cell function. Neural cell degeneration and damage can alter their blood levels. Higher NfL levels indicate more severe axonal degeneration, while more severe cognitive dysfunction correlates with lower GDNF levels. Previous studies have shown that plasma NfL levels are associated with cognitive function in PD patients (58) and can predict clinical conversion of cognitive function (59). It can also reflect changes of cognitive function level (60–62). Therefore, we adopted these indicators to assess acupuncture’s therapeutic effects. Since PD-MCI is difficult to diagnose due to its insidious onset and lack of specific biomarkers, this study also holds clinical value for exploring PD-MCI pathogenesis and improving diagnosis.

This study represents the first randomized, single-blind clinical trial investigating acupuncture treatment for cognitive dysfunction in PD. We employ a sham acupuncture device independently developed by our team as a control, thereby enhancing the credibility of evidence in acupuncture studies. Although some studies in acupuncture research have explored its therapeutic effects on cognitive impairment, no specific acupuncture intervention has yet targeted cognitive dysfunction in PD. Motor and non-motor symptoms in PD often interact and influence each other. Our study simultaneously assessed interventions for motor dysfunction, which may offer new insights into holistic management of PD. Additionally, the study explores the therapeutic mechanisms of acupuncture through clinical biofluid samples.

However, several limitations should be acknowledged. First, all enrolled cases are recruited from a single hospital, which may limit the generalizability of the findings across different regions. Second, minor stimulation may still occur in the sham group. Third, while patient blinding is successfully implemented, practitioners could not be blinded due to the nature of the acupuncture devices used in the study.

This study will provide reliable clinical evidence for the treatment of PD-MCI, thereby improving patients’ survival and quality of life while delaying or preventing its progression to PDD.
